# A robotic falcon induces similar collective escape responses in different bird species

**DOI:** 10.1098/rsif.2023.0737

**Published:** 2024-05-01

**Authors:** Rolf F. Storms, Claudio Carere, Robert Musters, Ronja Hulst, Simon Verhulst, Charlotte K. Hemelrijk

**Affiliations:** ^1^ Groningen Institute for Evolutionary Life Sciences, University of Groningen, Groningen, The Netherlands; ^2^ Department of Ecological and Biological Sciences, University of Tuscia, Viterbo, Italy; ^3^ Roflight, Enschede, The Netherlands

**Keywords:** RobotFalcon, deterrence, birds, collective escape, collective behaviour, predation

## Abstract

Patterns of collective escape of a bird flock from a predator are fascinating, but difficult to study under natural conditions because neither prey nor predator is under experimental control. We resolved this problem by using an artificial predator (RobotFalcon) resembling a peregrine falcon in morphology and behaviour. We imitated hunts by chasing flocks of corvids, gulls, starlings and lapwings with the RobotFalcon, and compared their patterns of collective escape to those when chased by a conventional drone and, in case of starlings, hunted by wild peregrine falcons. Active pursuit of flocks, rather than only flying nearby by either the RobotFalcon or the drone, made flocks collectively escape more often. The RobotFalcon elicited patterns of collective escape in flocks of all species more often than the drone. Attack altitude did not affect the frequency of collective escape. Starlings escaped collectively equally often when chased by the RobotFalcon or a wild peregrine falcon. Flocks of all species reacted most often by collective turns, second most often by compacting and third by splitting into subflocks. This study demonstrates the potential of an artificial aerial predator for studying the collective escape behaviour of free-living birds, opening exciting avenues in the empirical study of prey–predator interactions.

## 1. Introduction

Individuals of numerous species, including insects, fish, mammals and birds, frequently move collectively in an ordered fashion. Both grouping and collective motion aid in the reduction of predation risk: they decrease the chance per individual of being caught (‘dilution effect’ [1]), increase its odds of spotting the predator early (‘many eyes’ [1]), decrease the area individuals are at risk of being attacked from by a predator (‘selfish herd’ [2,3]) and help individuals to confuse the predator (‘confusion effect’ [1,4,5]). Under attack, bird flocks react with collective escape, that is, a series of coordinated motions of flock members resulting in specific patterns such as compacting, collective turns, wave events, flash expansions, cordons, splits and merges [6–10]. Empirical studies of patterns of collective escape of birds have mainly focused on starlings *Sturnus vulgaris*, because of their large flock size and their common and complex displays [11–14]. However, due to the difficulty of studying prey–predator interactions in the field, our understanding of collective escape behaviour in birds is still rudimentary.

Two major obstacles hamper the systematic and experimental study of prey–predator interactions under natural conditions: being present to observe when a predator attacks a flock, and the lack of control of the way in which the predator hunts the prey. Ethorobotics offers a solution to these obstacles, by enabling full control over artificial predators. This novel field of research has been proven successful in both studying predator–prey interactions in several species and providing solutions for ecological problems. It was shown in fish that ethorobotics can be used to control invasive species like mosquitofish: exposing mosquitofish in the wild to a robotic predator increased their fear and stress response during weeks after exposure, resulting in weight loss, altering body shape and lowering fecundity [15]. In locusts, robotic predators have been used to study surveillance and escape [16], and in birds, this approach was applied to study the fear response of individual birds [17].

To study patterns of collective escape, we therefore developed an artificial predator, the RobotFalcon [18]. We modelled the artificial predator after the peregrine falcon *Falco peregrinus*, because this raptor is a cosmopolitan aerial predator of many bird species, and hence suitable to study predator–prey interactions in many avian taxa. We have previously shown its effectiveness to clear fields from different species of birds within minutes [18], and here we have used the RobotFalcon for studying patterns of collective escape. Specifically, we compared how patterns of collective escape (1) differed among species, and depended on (2) the predator type (RobotFalcon or drone) and (3) the intensity of chasing actions by the predator. For the starlings only, we (4) compared flock responses between chases by the RobotFalcon and wild peregrine falcons [10,14].

## 2. Material and methods

### 2.1. Field work

**Figure 1.**
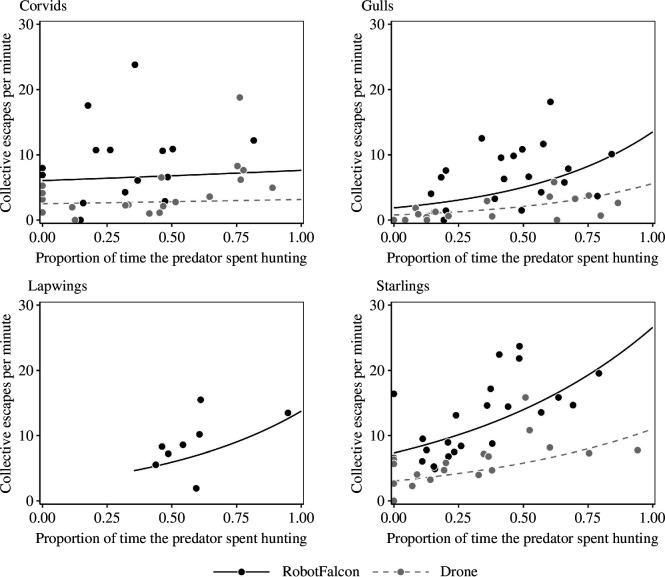
The effect of the proportion of time spent in active pursuit during chasing sequences by the RobotFalcon (66 chases) and drone (56 chases) on the frequency (min^−1^) of collective escape of flocks of corvids (34 chases), gulls (39 chases), lapwings (eight chases) and starlings (11 chases). Lines indicate the predictions made by the best-fitting regression model. With the exception of corvids, the higher the proportion of time the RobotFalcon or drone were actively chasing (by pursuit and/or attack of the flock) during a chasing sequence, the higher the frequency of collective escape exhibited by the flocks. See table 3 for the statistics.

We conducted fieldwork in the agricultural area surrounding Workum, The Netherlands (52°59′ N– 5°27′ E, figure 1), on 34 days between 25 February 2019 and 22 November 2019, excluding April– July to avoid disturbance to breeding birds. We chased with the RobotFalcon and drone on corvids (mixed flocks of *Corvus monedula*, *Corvus frugilegus* and *Corvus corone*), gulls (mixed flocks of *Chroicocephalus ridibundus* and *Larus canus*), northern Lapwings (*Vanellus vanellus*) and starlings. To minimize the impact of landscape on chasing, we confined our chases to flocks that were at least 100 m away from trees and buildings.

For a full description of RobotFalcon, drone and the field work protocol see Storms *et al*. [18]. Here, we use those same chasing events to investigate the collective responses of the ‘prey’. In brief, appearance of the RobotFalcon closely resembled a peregrine falcon in coloration, shape, overall size and the relative dimension of wing and tail. A DJI Mavic Pro drone lacking any raptor features was used for comparison. The field work area was searched by car, and when flocks were spotted on the ground, we recorded the species and number of individuals. The RobotFalcon and the drone were assigned randomly for chasing actions. Either ‘predator’ approached the flock until the flock took off. Flight initiation of a flock was defined as the start of a chase and a chase ended when the flock was out of sight (always the case with the RobotFalcon) or after 5 min (the drone did not always deter the flock, see [18]). Two certified operators (R.M. and R.W.) steered the RobotFalcon and the drone alternatingly. Prior to our chases, we recorded the speed and direction of the wind, using an anemometer (Kaindl windmaster 2) and a compass (Compass Galaxy). We did not chase during rain or strong wind (> 6 on the Beaufort scale).

The RobotFalcon approached flocks of ‘prey’ at a constant altitude, until they initiated flight. Approach altitude was either high (> 50 m) or low (< 50 m), randomly determined with a probability of 0.5. Once the flock was airborne, the pilot mimicked the hunting behaviour of real peregrine falcons [10,19] (table 1) by chasing the flock (pursuit), while occasionally ‘intercepting’ individuals by diving in the flock (attacks).

**Table 1.** Behavioural acts of the predator (RobotFalcon and drone) and patterns of collective escape by the flock.

behaviour/collective event	description
behaviour of predator (RobotFalcon and drone)	
attack	a manoeuvre approaching the flock aimed at intercepting prey (hunting)
pursuit	actively chasing the flock but not intercepting it
not actively chasing	flying in the vicinity of but not towards the flock
pattern of collective escape of flock	
collective turn	the flock changes direction with a minimum of 90°
compacting	the nearest neighbour distance decreases
split	single flock splits into multiple subflocks
collective dive	the flock flies downwards
merge	multiple subflocks merge together
blackening	the flock, or a part of it, darkens
flash expansion	birds suddenly move radially outward from the flock
cordon	two relatively large parts of the flock are interconnected by a thin string of individuals
wave event	one or more pulses of optically darkened bands propagating through the flock [14]

We recorded the behaviour of the birds with a ground camera (Sony FDR-AX53 4K Camcorder, 50 fps), supported by a camera on top of the RobotFalcon (Runcam micro swift2, 30 fps) and audio recordings by the observer (R.F.S.).

### 2.2. Data collection and analysis

We analysed the footage from the ground camera on a frame-by-frame basis and documented the type and frequency (per minute) of events of collective escape of flocks. We verified with the on-board camera on the RobotFalcon and drone the time points at which they were actively chasing the flock or not.

An event of collective escape was defined as a continuous period during which flock members collectively coordinate their motion to escape from the (artificial) predator. We based our classification of the patterns of collective escape on earlier analyses of starling flocks when hunted by peregrine falcons [10,14]. For instance, starling flocks regularly darken and exhibit wave events when hunted. Wave events are rapidly propagating dark bands which originate close by the predator, decrease predation success [14] and are likely caused by individual birds performing a zigzag manoeuvre [20]. Other patterns of collective escape include splits, during which flocks divide into two or more subflocks, merges of subflocks, flash expansions, which consist of birds moving radially outward from a central position in a flock and cordons, thin strings of individuals that connect two relatively large flock compartments [10]. As new patterns, we classified collective turns and collective dives. In table 1 and in the electronic supplementary material, videos, we report details for a complete description of the patterns of collective escape.

We defined active pursuits of the RobotFalcon and drone as actions during which they chased flocks (pursuits) with manoeuvres aimed at intercepting prey. Not actively pursuing was defined as the RobotFalcon or drone flying in the vicinity of flocks but not towards them (table 1).

We used generalized linear mixed models to analyse variation in the number of collective escapes exhibited by flocks (using the ‘glmer’ function of the lme4 package and the ‘ANOVA’ and ‘compareCoefs’ functions of the car package in R; [21]), including operator and flight_ID as random effects. Explanatory variables considered were target species, predator type (RobotFalcon or drone), chasing intensity (proportion of time the predator actively chased the flock), the presence of more species (yes/no) and the duration of the flight. We also tested whether there were significant interactions of target species with chasing intensity and predator type, and between predator type and chasing intensity. We assumed the response variable (the number of collective escapes) to be Poisson distributed and confirmed that the data were not overdispersed. Subsequently, we carried out a post hoc analysis for species differences in collective escapes (using the ‘emmeans’ function of the emmeans package in R). For starling flocks only, we tested whether the number of collective escape responses per chase differed significantly when chased by the RobotFalcon or by the real peregrine falcon. For this, we used data from the RobotFalcon presented in the current paper and from hunts by wild peregrine falcons collected earlier on starlings at urban roosts in Rome [10,22].

We also tested with generalized linear mixed models (using the ‘glmer’ function of the lme4 package) how the number of collective escape responses exhibited by flocks per 20 s after flight initiation was affected by the approach altitude of the RobotFalcon (> 50 or < 50 m).

Finally, we investigated the sequence of the types of collective escape patterns of the prey and the attacks by the predator. As in a previous analysis on starling flocks [10], a behaviour was classified to follow another event or behaviour when it was displayed within an interval of 5 s after that event or behaviour. To determine whether transitions occurred significantly more or less often than expected by chance we used a two-tailed permutation test, comparing against 100 000 matrices of random transitions generated using Patefield’s algorithm [23]. All statistical analyses were performed using R [21].

## 3. Results

Our dataset comprised 64 chases on bird flocks with the RobotFalcon, 49 chases with the drone and 46 hunts by real peregrine falcons on starlings in Rome (table 2). We recorded flocks escaping collectively from the RobotFalcon 707 times, from the drone 313 times and starling flocks escaping a real peregrine falcon 452 times.

**Table 2.** The number of chases performed by the RobotFalcon, drone and real peregrine falcon, their duration and the number of collective escape patterns exhibited by flocks of corvids, gulls, starlings and lapwings.

	chases (no.)	duration (s, mean ± s.e.)	collective escape patterns (no.)
RobotFalcon			
corvids	15	58.8 ± 9.7	113
gulls	20	67.6 ± 11.0	150
lapwings	8	70.4 ± 9.6	76
starlings	23	80.2 ± 8.0	368
total	66	70.3 ± 5.0	707
drone			
corvids	19	102.3 ± 19.3	89
gulls	19	195.8 ± 33.0	86
lapwings	—	—	—
starlings	18	95.2 ± 11.3	146
total	56	131.7 ± 14.6	313
peregrine falcon			
corvids	—	—	—
gulls	—	—	—
lapwings	—	—	—
starlings	46	—	452
total	46	—	452

**Figure 2.**
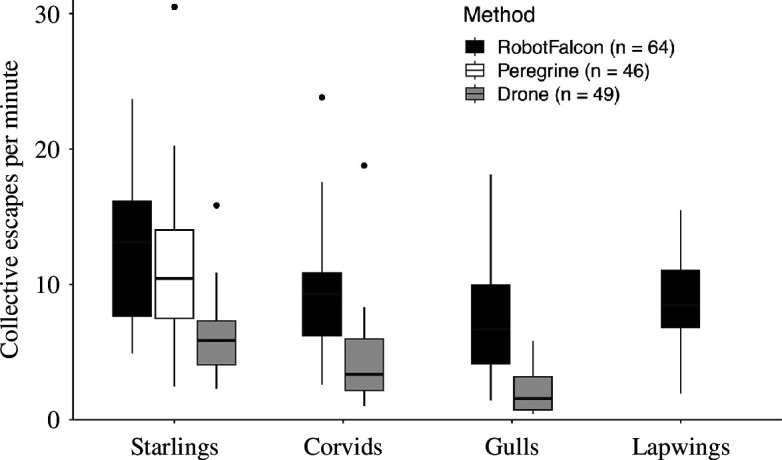
Boxplot of the number of collective escapes per minute for each species in response to the RobotFalcon, a real peregrine falcon (*F. peregrinus*) and a drone.

The rate of collective escapes (min^−1^) depended on the behaviour of the artificial predator: when the RobotFalcon or drone spent a larger proportion of time pursuing flocks actively (i.e. pursuing and attacking flocks), all patterns of collective escape were observed more frequently (figure 1; table 3). Furthermore, flocks of all bird species displayed patterns of collective escape more often when approached by the RobotFalcon compared to a drone (figures 1 and 2; table 3).

**Table 3.** Poisson generalized mixed model of the number of collective escapes in flocks of corvids, gulls, lapwings and starlings chased by the RobotFalcon and drone. Significant effects are highlighted in bold.

	estimate ± s.e.	chi-squared	*p* (> chi-squared)	
(intercept)	0.778 ± 0.272			
species		66.9012	<0.001 ***	
gulls	−1.390 ± 0.370			
lapwings	−0.932 ± 0.768			
starlings	0.353 ± 0.294			
predator_type		40.4128	<0.001 ***	
RobotFalcon	1.002 ± 0.273			
chasing_intensity	0.483 ± 0.411	27.5707	<0.001 ***	
duration_flight	0.00441 ± 0.000852	26.7659	<0.001 ***	
multiple_species		1.1860	0.276	
species:chasing_intensity		8.6811	0.033 *	
species:predator_type		4.5328	0.103	
predator_type:chasing_intensity		0.9945	0.319	
random factors		variance		
chase_ID		0.1808		
operator		8.481 × 10^−08^		

*Notes:* * indicates a significant effect with *p* < 0.05, ** indicates a significant effect with *p* < 0.01, and *** indicates a significant effect with *p* < 0.001.

**Figure 3.**
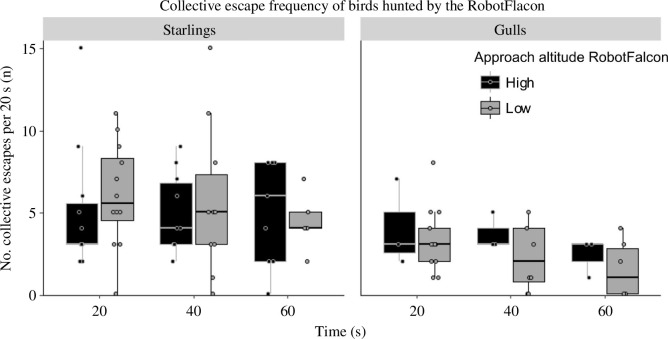
Boxplot of the number of collective escapes per 20 s by flocks of starlings and gulls in relation to approach altitude by the RobotFalcon and the time after flight initiation (binned per 20 s). High altitude: > 50 m, low altitude: < 50 m. Results for lapwings and corvids, for which there are fewer data, are shown in electronic supplementary material, figure S1.

The frequency of collective escape from each artificial predator differed significantly between species (figure 2; table 3). This was due to starlings exhibiting a higher frequency of collective escape than all the other species, significantly more than corvids and gulls, and due to corvids displaying significantly more collective escapes than gulls (figure 2; table 4). Whether the RobotFalcon approached flocks at high or low altitude did not significantly affect the frequency of collective escape responses (figure 3, electronic supplementary material, table S1).

**Table 4.** Post hoc analysis of species differences in the number of collective escapes. This post hoc analysis was performed on the best regression model from table 3. The estimated marginal means concern the average number of escapes per chasing sequence estimated from the model. Significant effects are highlighted in bold.

estimated marginal means of species		
species	estimated marginal means ± s.e.	
corvids	5.92 ± 0.631	
gulls	3.54 ± 0.426	
lapwings	4.32 ± 1.520	
starlings	10.69 ± 0.960	
**pairwise differences between species**		
pairwise comparison	*z*-ratio	*p*
corvids–gulls	3.247	0.0064 **
corvids–lapwings	0.862	0.8245
corvids–starlings	−4.795	<0.001 ***
gulls–lapwings	−0.540	0.9493
gulls–starlings	−7.719	<0.001 ***
lapwings–starlings	−2.518	0.0572

The number of collective escapes of starlings per chase when chased by the RobotFalcon and wild peregrine falcons did not differ significantly (*t*(67) = 1.11, *p* = 0.27; figure 2).

**Figure 4.**
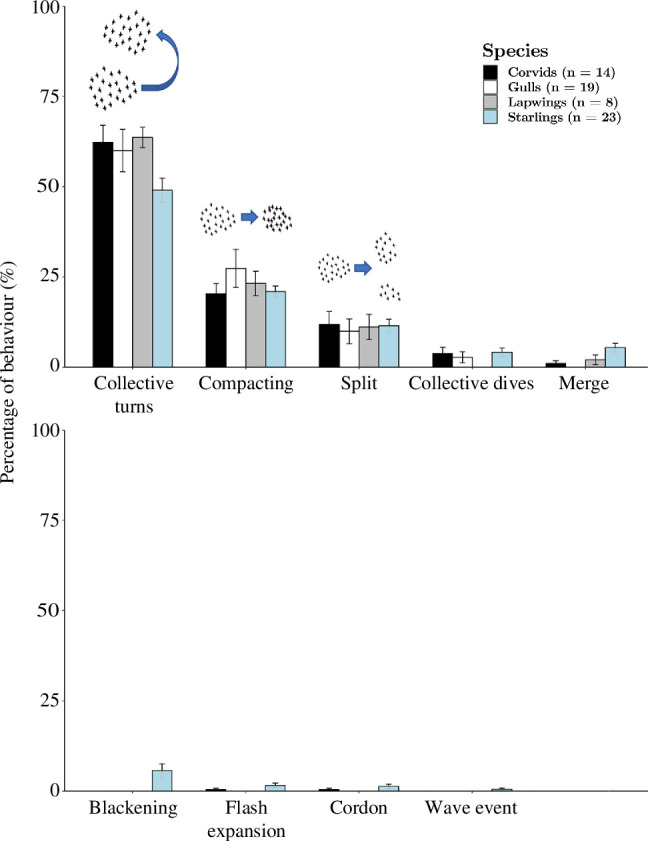
Distribution of different types of collective escape per species in response to the RobotFalcon. For each type of collective escape, the average percentage of the total number of collective escapes per chase has been plotted with standard errors.

The patterns of collective escape from the RobotFalcon in flocks of all four species consisted mostly of collective turning (49–64%), next most often of compacting (20–27%) and least frequently of splitting into subflocks (10–12%; figure 4). Other patterns of collective escape each comprised less than 6%.

**Figure 5.**
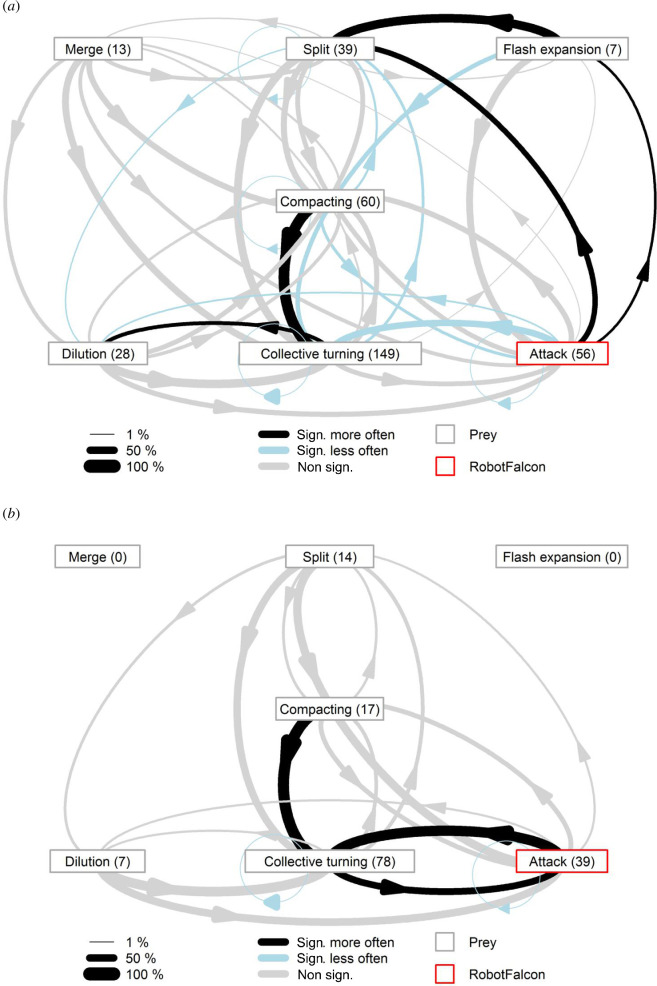
The transitions of collective escape in flocks of (*a*) starlings chased by the RobotFalcon (23 chasing sequences) and (*b*) gulls chased by the RobotFalcon (20 chasing sequences). See electronic supplementary material for corvids and lapwings.

Transitions between patterns of collective escape in flocks under predation were numerous, with each pattern of collective escape sharing a high connectivity to other patterns of collective escape (figure 5). However, flocks of all species showed collective turns after compacting significantly more often than predicted by chance. Attacks on starling flocks were significantly more likely followed by a flash expansion followed by a split than expected by chance (figure 5a). In gulls, attacks were significantly more often followed by collective turns than by other patterns of collective escape (figure 5*b*). Sample size was low for a meaningful analysis of transitions in corvids and lapwings (electronic supplementary material, figure S2).

## 4. Discussion

Studying predator–prey interactions in the wild is difficult because observing such interactions can be prohibitively time-consuming, and more importantly, neither prey nor predator is under experimental control. We resolved this by introducing an artificial predator under remote human control, the RobotFalcon. A critical question in this context is whether the prey perceived the RobotFalcon as a real predator, because only in this case will they behave accordingly. This question can only be addressed indirectly, by comparing the reactions of the prey to the RobotFalcon with those to a real peregrine falcon.

We have previously shown that the RobotFalcon is more effective in clearing fields from birds than a drone (a drone does not resemble a real predator), and that the flocks generally responded strongly to the RobotFalcon, without signs of habituation [18]. However, the fleeing behaviour of flocks in response to the RobotFalcon does not imply that they perceived the predator as real. Yet, in the case of flocks of starlings the frequency of collective escape to the RobotFalcon resembled that of the real peregrine falcon. This result supports the assumption that starlings perceived the RobotFalcon as a real predator. Note that this happened despite the difference in conditions between attacks by the RobotFalcon and the peregrine falcon: the starlings hunted by the real peregrine falcon were gathering to roost, whereas the starlings chased by the RobotFalcon were at their feeding site.

Do birds perceive differences in levels of threat [10,24]? We show that chasing with either the RobotFalcon or the drone was more likely to induce collective escape in flocks than flying in the vicinity of the flock without attacking (figure 1), and that the RobotFalcon elicited a higher frequency of collective escape than the drone. This confirms that birds increase their escape behaviour depending on the appearance and behaviour of the predator [25,26,27]. Similarly, earlier studies have shown that the pulses of agitation that cause wave events in starling flocks tend to weaken among individuals at a larger distance from the falcon [28]. On a functional level, it may be beneficial to save energy in less threatening situations by responding less intensely [29,30].

We have shown previously that a higher altitude of approach by the RobotFalcon induced earlier flight initiation [18]. This could indicate that the predator approaching from above was perceived as more threatening. However, in the current study, the altitude of approach did not affect the frequency of collective escape after flight initiation. This supports the alternative hypothesis that approaches by the predator from a high altitude are detected earlier, but do not represent a higher potential threat. Aspects of the experimental protocol may however also play a role. Specifically, the variation in approach altitude (high versus low) was often short-lived, as the RobotFalcon pursued the birds immediately after taking flight, at the altitude of the flock, limiting the opportunity to detect the effects of approach altitude on collective responses.

Little is known about the resemblance of patterns of collective escape among bird species. The studied species differ in morphology, in particular body size and wing shape. Despite these differences, we show that the different species resemble each other in the relative frequency of the different patterns of collective escape: collective turning happened most frequently, compacting second most often and splitting into subflocks third most often (figure 4). Similar selection pressures could have led to the evolution of similar collective escape in different bird species.

While collective escape patterns had similar relative frequencies in different species, there were clear differences in their absolute frequencies. Starlings in particular displayed collective escape more often than the other species (figure 2). This may be due to their smaller size, making them more vulnerable to threats and better at performing aerial manoeuvres [31]. Moreover, their flocks were typically larger in size than those of the other species: larger groups require a smaller proportion of informed individuals to flee, which could translate into a higher frequency of collective escape in the context of predation [32,33].

We showed that specific patterns of collective escape were related to each other and that attacks of the RobotFalcon led to the initiation of flash expansion and flock splitting significantly more often than expected by chance. This is in agreement with earlier findings from hunts by real peregrine falcons [10], and confirms that a robotic predator elicits a temporal structure of collective escape that is similar to that in response to real predators. Compacting also led to collective turning significantly more often than expected by chance, but understanding how and why this pattern arises requires further study.

Summarizing, we demonstrated that an artificial predator, the RobotFalcon, can be used for controlled experiments on predator–prey interactions in birds such as studying the consequences of approaches at different altitudes. This indicates the value of ethorobotics in studying complex ecological systems that have been seldom studied in the field. With respect to future work with the RobotFalcon, experiments can be expanded with other hunting strategies, such as the surprise attacks and repeated attacks described in wild peregrine falcons at starling roosts [19]. Other exciting research developments may involve tracking individual flock members, for example, through multiple cameras [34,35] and GPS loggers [8,9], and studying the effects of individual differences in, for example, personality using artificially composed flocks. In conclusion, there is great potential for the use of technologically advanced artificial predators, such as the RobotFalcon, to obtain a better understanding of the fascinating patterns of collective escape that characterize interactions between predators and flocks of prey.

## Data Availability

The data that support the findings of this study are uploaded to the Zenodo Research Data repository and are available online [36]. Electronic supplementary material is available online [37].
